# Rapid identification of Brucella sepsis/osteomyelitis in a 6-year old febrile patient with matrix-assisted laser desorption/ionization time-of-flight mass spectrometry directly from positive blood culture: a case report

**DOI:** 10.1186/s12879-019-3864-z

**Published:** 2019-03-11

**Authors:** Junfei Guo, Weiming Lai, Biting Li, Lingling Tang, Yongbing Wu, Yasha Luo, Chengyi Liu, Weiming Lu, Xiaoping Mu

**Affiliations:** Clinical laboratory department of Guangdong Women and Children’s hospital, Xiaoping Mu, No.521 Xingnan Road, Panyu, 511400 Guagnzhou China

**Keywords:** *Brucella*, Brucellosis, MALDI-TOF SP, Rapid identification

## Abstract

**Background:**

*Brucella* is high-consequence pathogen and one of the most common seen laboratory- acquired infection pathogens. Quick and accurate detection of the pathogen will be of great important to reducing laboratory- acquired infection. Traditional biomedical reaction based method is time consumption, and mass spectrometry based method greatly reduces time consumption in pathogen identification. In the case presented here, we shared our experience in identification of *Brucella* directly from positive blood culture with mass spectrometry based method.

**Case presentation:**

The patient is a 6-year boy with a history of three weeks fever accompanied with sweating and a pain at right patella. The patient also has a history of thalassemia and blood transfusion was performed previously admitted to our hospital. Two bottles of marrow culture and one bottle of blood culture were positive, and direct mass spectrometry from positive culture material revealed *Brucella* infection within 1 h.

**Conclusion:**

Clinical characters and laboratory findings of the patient presented here might help clinician in non-endemic region to made suspected brucellosis diagnose. Our experience in rapid identification of *Brucella* from positive blood culture with MALDI-TOF SP could help preventing laboratory-acquired infection of *Brucella*.

## Background

As a wide spread zoonosis, brucellosis remains a public health problem around the word. *Brucella spp.* is the causative pathogen of the disease, and *B.melitensis* and *B. abortus* are the most common isolated species [1]. The diagnosis of the disease depends on the isolation of the pathogen, which needs several days with previous biochemical reaction based identification methods. As no enough precautions were paid to these high-consequence pathogens at routine clinical laboratory especially in non-endemic areas, it causes one of the most prominent laboratory-acquired infections around the world [2, 3]. Thus quick and correct identification of the pathogen will be of great interest in reducing the laboratory-acquired infections of *Brucella* [4, 5].

Here, we shared our experience in a recent case of brucellosis. We identified the pathogen directly from positive blood and marrow culture with Bruker MALDI-TOF within 2 h after blood culture became positive. It’s of great interest considering that our blood culture bottle is BACT/ALERT® PF with activated charcoal, which is considered not suitable for directly identified with MALDI-TOF. We also shared our two-step centrifugation ethanol/formic acid tube extraction method, which we think will be of great help in case of lacking of MBT Sepsityper Kit or in the situation like us use Bact/ALERT 3D blood culture system.

## Case presentation

A 6-year old boy with a history of 3-week fever (unknown origin) accompanied by weight lost (− 2.5 Kg) was admitted to pediatric department of Guangdong Women and Children Hospital. The boy experienced a 3-week of fever and sweating, which mainly happened at night and peaked at 40.3 C degree. A pain at right patella was also reported. What’s more, the boy had a history of thalassemia with hemoglobin fluctuating between 95 and 100 g/L. Blood transfusion and antibiotic treatment (Cefperazone-Sulbactam, Azithromycin and piperacillin-sulbactam) were conducted at a local hospital before admitting to our hospital, but intermittent fever continued. On admission, physical examination and laboratory detection were conducted. All the physical examinations were normal, except for diffuse enlargement of mesenteric lymph nodes. Laboratory test indicated a drop of white blood cell (3.33*10^9/L, N,26.7% L,64.65) and hemoglobin (71 g/L), and increase of erythrocyte sedimentation rate (ESR,25 mm/h) and ferroprotein (FER, 1669 ng/ml). Slight increase of ALT (105 U/L) and AST (145 U/L) and significant increase of LDH (2082 U/L) were also observed. The serum level of high sensitive c-response protein (hsCRP) was normal (6.81 mg/L), and the level of procalcitonin (PCT) was slight increase (0.16 ng/ml). At the time of admission, two sets of blood culture and two sets of bone marrow culture were obtained. Two marrow culture presented positive 2.6 days post obtained, and one set of blood culture present positive 3.6 days post obtained, while the other set of blood culture remained negative (5 days).

## Pathogen identification directly form positive blood culture with MALDI-TOF

Gram stain of smear prepared from positive blood culture was done, but no bacteria were seen throughout the slide (three of our laboratory workers viewed the slide). MALDI-TOF analysis directly from positive blood culture was conducted with two-step centrifugation method as previous reported with some modification. The procedures were as follow: 1.5 ml of positive blood/marrow culture was transferred to 1.5 ml centrifugation tube, and then centrifuged for 2mins at 500 rpm (eppendorf, centrifuge 5945R); the supernatant was transferred to a new tube, centrifuged for 2mins at 12,000 rpm and discarded the supernatant; re-suspended the precipitation with 1 ml sterile water, and centrifuged for 2mins at 12,000 rpm; the final precipitation was proceeded with standard methane acid extraction method. MALDI-TOF analysis yielded none reliable identification while searching standard database (IVD BD5989). Considering the clinical characteristic and laboratory findings of the patient, as well as long time consumption before blood/marrow culture became positive, we reanalyzed the spectrum with self-construct database (provided by the supplier) containing four isolates of *Brucella*. The result demonstrated that the spectrum hit two of four *Brucella* isolates with score of 1.90 and 1.88, respectively. Suspected *Brucella* infection was considered, and all the biomaterials of the patient were sealed immediately. A communication was made with clinician for bio-safety and epidemiology purposes.

## Pathogen confirmation with isolations by MALDI-TOF and Vitek2 compact system

All the isolates separated from positive blood/marrow culture presented morphology of *Brucella*. We used on-plate formic acid method to prepare the sample for MALDI-TOF analysis. Four reliable identifications of *Brucella* with score ranging from 2.3 to 2.4 were obtained. As our self-construct database could not identify the *Brucella* to species level, we re-identified the isolates with Vitek2 Compact system with GN card, and the results demonstrated that the isolates were all *B. melitensis* with 98% confidences level. Definite *Brucella* infection was confirmed, and the patient was transferred to specialized hospital for infectious diseases according to relative law.

## Discussion and conclusions

**Fig. 1:**
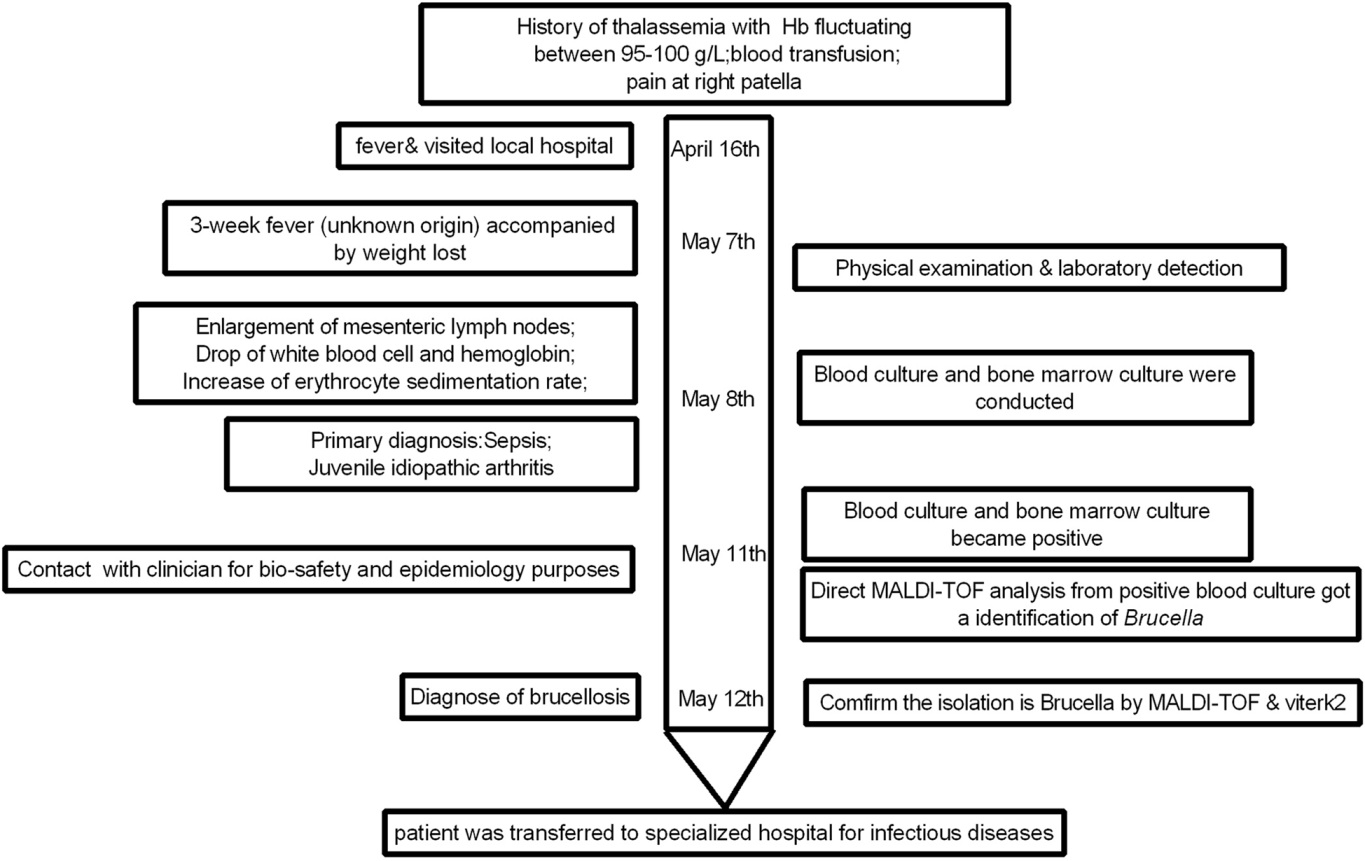
The timeline of our case The showing picture is the timeline of the reported case. Above the arrow is the medical history of the patient, the arrow represents the time course, the left side is the symptoms, signs and diagnosis along time, the right side is the course of pathogen identification

In the case presented here, we shared our experience in the use of MALDI-TOF for rapid identification of *Brucella* infection directly from positive blood culture (Fig. 1). Laboratory workers are at high risk of getting laboratory-acquired infection of *Brucella* via direct contact or exposure to aerosol [3–5]. Quick and correct identification will be great help in reducing thus infection. Compared with our previous reported case [6], we saved almost 48 h from positive blood culture to correct identification of pathogen. It’s of great interest considering that our hospital located at non-endemic area and no enough precautions are paid to thus high-consequence pathogen at our biosafety level (BSL)2 laboratory. Our rapid identification of the pathogen avoided more individuals exposed to the high-risk pathogen.

Many previous studies reported using MALDI-TOF for rapid identification of the bacteria from positive blood culture [7–9]. In many cases, they used BACTEC™ FX system for blood culture and use MBT Sepsityper Kit to prepare the sample for MALDI-TOF analysis. Positive blood culture with BACT/ALERT® PF is considered not suitable for direct MALDI-TOF analysis as activated charcoal may affect the analysis. In an on-going project, we find that two-step centrifugation ethanol/formic acid tube extraction method could yield good identification results, and the case presented here is an example. As many hospitals use Bact/ALERT 3D system for blood culture, our experience might help in rapid identification of the pathogen with Bruker MALDI-TOF while using Bact/ALERT 3D blood culture system.

In this case, we found that good quality spectrums were obtain both from positive blood culture or isolates, but yield no reliable identification while searching standard database (IVD BD5989) only. In fact Bruker provides security relevant database, but it’s restricted in some countries like China due to the local law. A self-construct database (provided by the supplier) helps us rapid identifying the pathogen. This self-construct database contains the spectrums of four *Brucella* isolates (two *B.melitensis* and two *B. abortus*). It’s of great help in identification less-common isolated pathogens using such self-construct database.

As the symptoms of brucellosis are nonspecific (mainly fever accompanied by sweating), it’s difficult to diagnosis of the disease from clinical sign only. Our patient visit a local hospital but the disease did not diagnose, and experienced antibiotic treatment was conducted. This may the reason why one of the blood cultures remained negative. Our rapid identification of the pathogen leads to prompt diagnosis of the disease. Combine with our previous reported case of brucellosis [6], we think that cautions should be paid to the unknown origin long-term fever patient with following laboratory results: A drop of WBC (dominant with lymphocyte), unchanged or slightly increase hsCRP or PCT level, slightly increase of ESR, anemia and sometimes enlargement of local lymph nodes. Directly identification of the pathogen from positive blood culture of these patients might help diagnose the disease ahead of time, which will be of great interest in preventing the spread of the disease in case of brucellosis.

In all, a good combination of blood culture and MALDI-TOF analysis will be of great help in preventing laboratory-acquired infection of high-consequence pathogens, such as *Brucella*. Our experience might help those with Bruker MALDI-TOF mass spectrometry but using Bact/ALERT 3D blood culture system or those without MBT Sepsityper Kit at hand.

## Abbreviations

FER: Ferroprotein
hsCRP: High sensitive c-response protein
MALDI-TOF SP: Matrix-assisted laser desorption/ionization time-of-flight mass spectrometry
PCT: Procalcitonin
